# Increased risk of malignancy in patients with Takayasu’s arteritis: a population-based cohort study in Korea

**DOI:** 10.1038/s41598-022-24324-0

**Published:** 2022-12-21

**Authors:** Seulkee Lee, Seonyoung Kang, Yeonghee Eun, Hyungjin Kim, Jaejoon Lee, Eun-Mi Koh, Hoon-Suk Cha

**Affiliations:** 1 Department of Medicine, Samsung Medical Center, Sungkyunkwan University School of Medicine, 81 Irwon-Ro, Gangnam-Gu, Seoul, 06351 Republic of Korea; 2 Division of Rheumatology, Department of Medicine, Kangbuk Samsung Hospital, Sungkyunkwan University School of Medicine, Seoul, Republic of Korea

**Keywords:** Rheumatology, Vasculitis syndromes

## Abstract

This study aimed to evaluate the relative risk of malignancy in patients with Takayasu’s arteritis compared to that in the general population. This retrospective nationwide cohort study used data from the Korean Health Insurance Review and Assessment Service database. All newly diagnosed patients with Takayasu’s arteritis were identified between January 2009 and December 2019. They were observed until the diagnosis of malignancy, death, or end of the observational period, December 2020. The standardized incidence ratios (SIRs) of the overall and site-specific malignancies were estimated and compared with the incidence of cancer in the general population retrieved from the National Cancer Registry. We identified 1449 newly diagnosed patients with Takayasu’s arteritis during the observational period (9196 person-years). A total of 74, 66, and 8 patients had overall, solid, and hematologic malignancies, respectively. The risks of overall [SIR, 1.62; 95% confidence interval (CI) 1.27–2.03], solid (SIR, 1.51; 95% CI 1.17–1.92), and hematologic (SIR, 4.05; 95% CI 1.75–7.98) malignancies were increased compared to those in the general population. In solid malignancies, breast (SIR, 2.07; 95% CI 1.16–3.42) and ovarian (SIR, 4.45; 95% CI 1.21–11.39) cancers had an increased risk. In hematologic malignancies, the risk of myelodysplasia increased (SIR, 18.02; 95% CI 3.72–52.66). Immunosuppressive agent use was not associated with malignancy. There was no specific period when cancer more frequently occurred. An increased risk of malignancy was observed in patients with Takayasu’s arteritis compared to that in the general population in this large-scale nationwide population study of Korean health insurance data.

## Introduction

Takayasu’s arteritis (TAK) is a chronic idiopathic inflammatory disease that primarily affects large vessels such as the aorta and its main branches^1,2^. It is prevalent among young Asian females and has an early onset between 10 and 40 years of age^3,4^. Due to vessel inflammation, complications such as wall thickening, fibrosis, stenosis, and thrombus formation occur^1^. However, the mortality rate of cardiovascular events has been decreasing due to treatment advances^5^.

Recent studies have identified neoplasms as the second most common cause of death in patients with TAK^6,7^. Various autoimmune diseases, including rheumatoid arthritis, systemic lupus erythematosus, Sjögren’s syndrome, systemic sclerosis, and inflammatory myositis, are related to the increased rates of malignancies^8–12^. However, the incidence of malignancies in systemic vasculitis is relatively unknown. Among systemic vasculitis, small-vessel vasculitis has been the most frequently studied. Several studies have demonstrated an increased risk of malignancy in anti-neutrophil cytoplasmic antibody-associated vasculitis (AAV)^13–15^. However, information regarding the risk of malignancies in patients with large-vessel vasculitis remains scarce. Despite conflicting data, most studies concluded that there is no increase in the risk of malignancies in giant cell arteritis (GCA)^16–18^. In the case of TAK, there have been few reports of malignancies^19–21^. However, only one study compared the risk of malignancies in TAK to that of the general population^22^. In a previous study, overall malignancies did not differ significantly between patients with TAK and the general population; however, the risk of myelodysplastic syndrome (MDS) was significantly increased. However, no conclusion could be drawn since only one MDS patient was included in that single-center study of 180 patients with TAK.

TAK is a rare disease with an incidence of approximately 1.11 per million person-years^23^; therefore, cohort studies have limited ability to reveal the risk of malignancies. Here, we evaluated the relative risk of malignancies in patients with TAK and compared them with the general population using the medical insurance data of South Korea.

## Methods

### Data acquisition

#### National claims database of Korea for TAK patients

The Health Insurance Review and Assessment Service (HIRA) database, which includes the healthcare utilization information of approximately 50 million South Koreans, was the primary data source of this study. The information is registered by the Korean National Health Insurance Service (NHIS), a mandatory nationwide health insurance system that covers virtually the entire population. It contains data regarding the individual beneficiaries and healthcare service information, such as diagnoses, procedures, prescriptions, and diagnostic tests from inpatient and outpatient care. Patients registered with the M31.4 (International Classification of Diseases (ICD)-10 code for TAK) code at least once between January 2007 and December 2020 were extracted for further analysis.

#### Incidence of malignancy in the general population

The incidence of malignancy in the general population was retrieved from the 2014 National Cancer Registry (NCR), which collects the annual incidence of malignancies in the entire population of South Korea by age, sex, and malignancy type.

### Study population

We identified all patients registered with a first-time diagnosis of TAK between 2009 and 2019. We selected patients with the M31.4 code as the primary disease code at least twice to reduce the risk of including misclassified cases of TAK. To obtain the M31.4 code as the primary disease code in South Korea, patients had to be registered in the rare intractable disease (RID) program. All patients in the RID program had to satisfy the American College of Rheumatology 1990 criteria for the classification of TAK. To ensure that the TAK diagnosis was new, only patients with an interval of less than 6 months between the first and second visits were included. In addition, a washout period of 2 years (2007–2008) was applied to exclude the previous prevalent cases. Furthermore, only patients diagnosed with TAK by December 2019 were included in the analysis to ensure a follow-up period of at least 1 year. The date of study inclusion (index date) was defined as the date of the first hospital contact for TAK.

This research complied with the principles of the Declaration of Helsinki. The institutional review boards (IRB) of Samsung Medical Center approved this study (SMC 2021–01-058). The database extracted from the NHIS could not be identified directly or through identifiers linked to the subject. Thus, the need for obtaining informed consent from the participants was waived by the IRB of Samsung Medical Center.

### Definition of incident malignancy

The occurrence of malignancy was regarded as the first claim under ICD-10 codes, including C00–97, D46, and D47.1, according to the incidence of malignancy classification published by the NCR. The date of the first claim of each malignancy was set as the date at which the diagnosis of malignancy was made. To ensure that malignancy occurred after TAK diagnosis, patients with malignancy before the index date were excluded from the analysis.

### Risk of malignancy based on medication usage

The risk of malignancy according to the use of medications was investigated. Prescriptions for glucocorticoids (prednisolone, methylprednisolone, deflazacort, dexamethasone, hydrocortisone, and triamcinolone), methotrexate, azathioprine, cyclophosphamide, mycophenolate mofetil, leflunomide, tacrolimus, cyclosporine, anti-tumor necrosis factor-alpha inhibitors, and tocilizumab were collected after the index date.

### Risk of malignancy based on comorbidities

The risk of malignancy according to comorbidities was investigated using the Charlson comorbidity index (CCI)^24^ (the list of comorbidities identified using the CCI is presented in Table S1). Based on a previous study that utilized the HIRA data^25^, comorbidities registered within 1 year from the index date were included in the analysis. We categorized the patients with low, medium, and high comorbidities as CCI 0–1, 2, and 3 or larger, respectively.

### Statistical analysis

We calculated the standardized incidence ratios (SIRs) of overall and site-specific cancers after dividing patients by age into 10-year intervals to compare the incidence of cancer in patients with TAK and the general population. The observed and expected number of cases were calculated for each age group using the age- and sex-specific incidence data. SIR was estimated by dividing the number of observed cancers by the number of expected cancers, and a 95% confidence interval (CI) was estimated using the Poisson distribution.

We calculated the number of expected cancer cases by multiplying the age-specific cancer incidence rate of the general population from the 2014 NCR and the person-years of patients with TAK. The types of malignancies were compared according to the NCR. In the subgroup analysis, Hodgkin lymphoma (C81), non-Hodgkin lymphoma (C82–86, C96), multiple myeloma (C90), leukemia (C91–95), MDS (D46), and myeloproliferative disease (D47.1) were combined as hematologic malignancies, whereas central nervous system (C70–72), gall bladder/cholangiocarcinoma (C23–24), salivary gland (C07–08), liver (C22), connective tissue or soft tissue (C47, C49), oral cavity (C03–06), thymus, heart, or pleura (C37–38), non-melanoma skin cancer (C44), bladder (C67), esophagus (C15), and unspecified were combined as “Others” due to the small number of patients in each malignancy.

A Cox proportional hazard analysis was performed to determine whether medication use affected the occurrence of malignancy. Medication usage was analyzed as a time-varying variable during the follow-up period. In this analysis, the status of each medication use changed over time. The follow-up period started on the index date, which was similar to that of the SIR analysis. In the Cox proportional hazard analysis, the age, sex, and index date of TAK diagnosis were included as covariates to adjust for the effect of covariates on the development of malignancy. The hazard ratios (HRs) and corresponding 95% CIs were calculated using the Cox proportional hazard model.

Categorical variables were presented as frequency (%) and compared using the chi-square test. Continuous variables were presented as a mean (standard deviation) and analyzed using the Student’s *t*-test. Values of *p* < 0.05 (two-sided) were considered statistically significant. R software (version 3.6.3, R Foundation for Statistical Computing, Vienna, Austria) was used for all statistical analyses.

## Results

### Baseline characteristics

**Figure 1:**
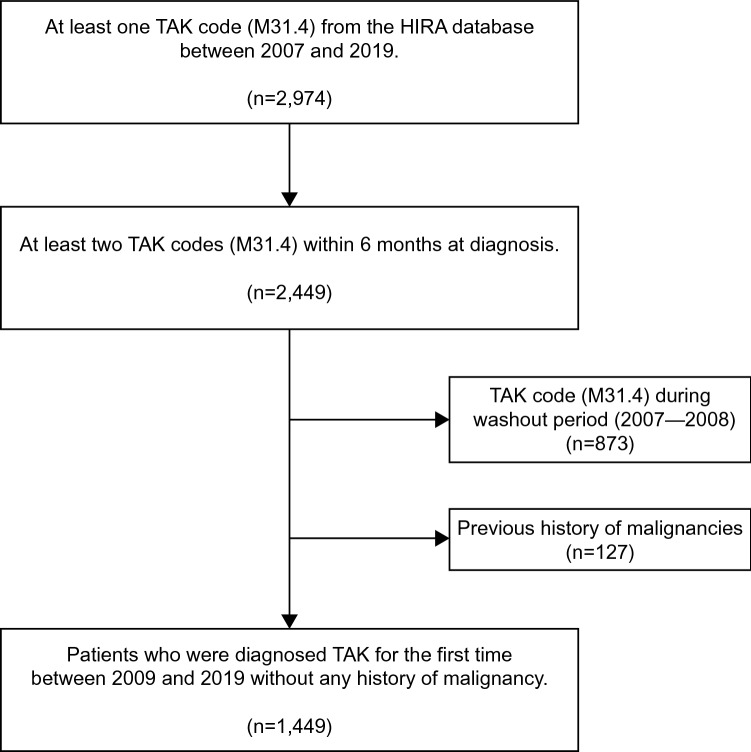
Flowchart of the selection process of included patients with Takayasu’s arteritis.

Among the 2974 patients with TAK extracted from the HIRA database, 2449 had at least two M31.4 codes within 6 months, whereas 873 had the M31.4 code during the washout period. A total of 127 patients with a history of malignancies were excluded. We finally identified 1449 patients who were newly diagnosed with TAK between 2009 and 2019 for further analysis (Fig. 1).

The clinical characteristics of TAK patients are shown in Table 1. Approximately 81.1% of patients were women, and the mean age of the cohort at the diagnosis of TAK was 47.00 ± 15.56 years. The mean duration of observation was 6.35 ± 3.23 years. A total of 702 (48.4%) patients used glucocorticoids. The most frequently used immunosuppressive agent was methotrexate (n = 469, 32.4%), followed by azathioprine (n = 237, 16.4%). The clinical characteristics of patient groups according to the presence or absence of a malignancy are presented in Table S2 (Additional File 1). TAK patients with malignancy were older at the time of diagnosis of TAK than those without malignancy (50.93 ± 15.36 vs. 46.79 ± 15.55 years, *p* = 0.026). The mean duration from the diagnosis of TAK until the development of malignancy was 3.38 ± 2.86 years. The use of immunosuppressive agents was not significantly different between the two groups.

**Table 1:** Clinical characteristics of the study population.

	All
n	1449
Sex, female	1175 (81.1)
Age at diagnosis of TAK, years	47.00 ± 15.56
Duration of observation, years	6.35 ± 3.23
**Medication**	
Glucocorticoids	702 (48.4)
Methotrexate	469 (32.4)
Azathioprine	237 (16.4)
Leflunomide	20 (1.4)
Cyclophosphamide	12 (0.8)
Mycophenolate mofetil	27 (1.9)
Tacrolimus	36 (2.5)
Cyclosporine	31 (2.1)
Anti-TNF	17 (1.2)
Tocilizumab	7 (0.5)
**CCI**	
0	286 (19.7)
1	407 (28.0)
2	328 (22.6)
3≤	428 (29.5)

Numerical quantitative data are presented as mean ± standard deviation.

Categorical data are presented as frequency (%).

TAK*,* Takayasu’s arteritis; TNF*,* tumor necrosis factor; CCI, Charlson comorbidity index.

### Increased risk of malignancy in patients with TAK versus the general population

During the observation period (9196 person-years), 74 overall malignancies occurred in patients with TAK (Table 2). Among them, 66 were solid malignancies and 8 were hematologic malignancies. The breast (n = 15) was the organ with the highest number of site-specific malignancy cases, followed by the thyroid (n = 14) and lung (n = 8). MDS was the most prevalent hematologic malignancy (n = 3).

**Table 2:** Comparison of malignancy type and sex by number, expected incidence, and standardized incidence ratio compared with general population.

Type of malignancy	Total (n = 1449; 9196 PYs)			Female (n = 1175; 7571 PYs)			Male (n = 274; 1626 PYs)		
	n	Expected^¶^	SIR (95% CI)	n	Expected^¶^	SIR (95% CI)	n	Expected^¶^	SIR (95% CI)
All	74	45.69	1.62 (1.27–2.03)	55	35.91	1.53 (1.15–1.99)	19	9.78	1.94 (1.17–3.03)
Solid malignancy	66	43.71	1.51 (1.17–1.92)	52	34.43	1.51 (1.13–1.98)	14	9.28	1.51 (0.82–2.53)
Breast (C50)	15	7.23	2.07 (1.16–3.42)	14	7.23	1.94 (1.06–3.25)	1	0.01	151.43 (3.83–843.69)
Thyroid (C73)	14	9.70	1.44 (0.79–2.42)	11	9.24	1.19 (0.59–2.13)	3	0.46	6.55 (1.35–19.15)
Lung (C33–34)	8	3.68	2.18 (0.94–4.29)	4	2.23	1.80 (0.49–4.60)	4	1.45	2.75 (0.75–7.05)
Uterine	4	2.22	1.80 (0.49–4.61)	4	2.22	1.80 (0.49–4.61)	NA		
Cervix (C53)	2	1.23	1.63 (0.20–5.89)	2	1.23	1.63 (0.20–5.89)	NA		
Corpus^†^ (C54)	2	0.93	2.16 (0.26–7.80)	2	0.93	2.16 (0.26–7.80)	NA		
Ovary (C56)	4	0.90	4.45 (1.21–11.39)	4	0.90	4.45 (1.21–11.39)	NA		
Stomach (C16)	4	4.91	0.82 (0.22–2.09)	3	3.11	0.96 (0.20–2.81)	1	1.79	0.56 (0.01–3.11)
Kidney (C64)	3	0.76	3.96 (0.82–11.58)	2	0.49	4.06 (0.49–14.67)	1	0.26	3.78 (0.10–21.05)
Others^‡^	14	5.97	2.35 (1.28–3.94)	10	3.75	2.67 (1.28–4.90)	4	2.22	1.81 (0.49–3.94)
Hematologic malignancy	8	1.98	4.05 (1.75–7.98)	3	1.48	2.03 (0.42–5.94)	5	0.50	10.00 (3.25–23.33)
MDS (D47.1)	3	0.17	18.02 (3.72–52.66)	1	0.12	8.45 (0.21–47.06)	2	0.05	41.58 (5.04–150.19)
Myeloid leukemia (C92–94)	2	0.37	5.45 (0.66–19.69)	1	0.27	3.68 (0.09–20.50)	1	0.13	7.52 (0.19–41.87)
Hogkin lymphoma (C81)	1	0.04	24.91 (0.63–138.81)	1	0.03	35.09 (0.89–195.51)	0	0.01	0.00 (0.00–316.88)
Non-Hogkin lymphoma (C82-C86, C96)	1	0.74	1.36 (0.03–7.56)	0	0.55	0.00 (0.00–6.75)	1	0.19	5.24 (0.13–29.21)
Multiple myeloma (C90)	1	0.28	3.56 (0.09–19.82)	0	0.21	0.00 (0.00–17.31)	1	0.07	14.68 (0.37–81.79)

^¶^Expected number of patients with malignancy calculated based on the incidence of malignancy in the age- and sex- matched general population.

^†^Corpus uterine cancer includes malignancies in endometrium, myometrium, and fundus.

^‡^CNS (C70–72), GB/CCC (C23–24), salivary gland (C07–08), liver (C22), connective tissue or soft tissue (C47, C49), oral cavity (C03–06), thymus, heart, or pleura (C37–38), non-melanoma skin cancer (C44), bladder (C67), esophagus (C15), and unspecified.

*PYs* person-years, *SIR* standardized incidence ratio, *MDS* myelodysplastic syndrome, *CNS* central nervous system, *GB* gall bladder, *CCC* cholangiocarcinoma.

The risk of overall malignancies combined was increased in patients with TAK (SIR, 1.62; 95% CI 1.27–2.03), and the risks of both solid (SIR, 1.51; 95% CI 1.17–1.92) and hematologic malignancy (SIR, 4.05; 95% CI 1.75–7.98) were also increased (Table 2). Among the risk of solid malignancy, breast cancer (SIR, 2.07; 95% CI 1.16–3.42) and ovarian cancer (SIR, 4.45; 95% CI 1.21–11.39) demonstrated significantly higher risks in patients with TAK than in the general population. Regarding the risk of hematologic malignancy, only the risk of MDS was increased (SIR, 18.02; 95% CI 3.72–52.66).

The overall malignancy incidence was higher in both sexes (female: SIR, 1.53; 95% CI 1.15–1.99; male: SIR, 1.94; 95% CI 1.17–3.03) in patients with TAK than in the general population. However, when the malignancies were categorized into solid and hematologic types, the risk of solid malignancies increased in females (SIR, 1.51; 95% CI 1.13–1.98), whereas the risk of hematologic malignancies increased in male patients with TAK (SIR, 10.00; 95% CI 3.25–23.33).

### Risk of malignancy based on medication usage

We investigated the effect of medications on the occurrence of malignancies in patients with TAK. The effects of glucocorticoids, methotrexate, and azathioprine were analyzed. The age at the diagnosis of TAK, sex, and index year were used as covariates to adjust for the effects of these factors. The use of long-term glucocorticoids (≥ 1 year), methotrexate, and azathioprine was not independently associated with the development of malignancies (Additional File 1, Table S3).

### Risk of malignancy based on comorbidities

Because we could not obtain information on comorbidities from the general population, the incidence of malignancies could not be determined by adjusting for comorbidities in patients with TAK and the general population. Instead, the SIRs of malignancies for patients with TAK with low (CCI 0–1), medium (CCI 2), and high comorbidities (CCI ≥ 3) were calculated and compared with those of the general population (Additional File 1, Table S4). The risk of overall malignancies was significantly higher in patients with TAK with medium (SIR, 1.86; 95% CI 1.10–2.94) and high comorbidities (SIR, 1.81; 95% CI 1.22–2.58) compared with that in the general population. However, the risk of overall malignancies was not significantly different between patients with TAK with low comorbidity (SIR, 1.35; 95% CI 0.88–1.97) and in the general population.

### Cumulative incidence of malignancy during the observation period

The cumulative incidence of malignancy during the observation period in patients with TAK was evaluated. Since this study selected newly diagnosed patients during the observation period, cumulative incidence provided information regarding when the malignancy occurs most frequently after the diagnosis of TAK. However, malignancies steadily occurred throughout the observation period (Additional File 2). Thus, a clear temporal relationship between the diagnosis of TAK and malignancy was not observed.

### Relative risk of malignancy stratified by the year of TAK diagnosis

To determine the difference in the risk of malignancy according to the year of diagnosis, we divided the patients into three groups by the year of TAK diagnosis: 2009–2012, 2013–2016, and 2017–2019. The risk of overall malignancies was significantly higher in patients with TAK diagnosed between 2013–2016 (SIR, 1.81; 95% CI 1.22–2.58; Additional File 1, Table S5) and 2017–2019 (SIR, 2.50; 95% CI 1.33–4.27) than in the 2009–2012 group. However, the risk of overall malignancies was not significantly different in patients diagnosed with TAK between 2009 and 2012 compared with that in the general population.

### Sensitivity analysis using the second hospital visit as the index date

Concerned that an immortal time bias would occur when the date of the first hospital visit was used as the index date, sensitivity analysis was performed using the date of the second visit as the index date. In this analysis, compared with the general population, the risk of overall malignancies combined was increased in patients with TAK (SIR, 1.59; 95% CI 1.25–2.00), and the risk of both solid (SIR, 1.48; 95% CI 1.14–1.89), along with an increase in hematologic malignancies (SIR, 4.09; 95% CI 1.77–8.06), which was similar to the results of the original analysis.

## Discussion

In this study, we demonstrated that the risk of malignancy in patients with TAK was higher than that in the general population. The most common solid malignancy was breast cancer, followed by thyroid and lung cancers. In women, breast and ovarian cancers were more common than in the general population, and in men, thyroid cancer was more common. On the contrary, MDS was the most common in hematologic malignancy. The risk of hematologic malignancy increased in TAK patients, but not in females. Among the hematologic malignancies, only the risk of MDS was higher in male TAK patients than in the general population. Immunosuppressive agent use was not significantly associated with the risk of malignancy.

This is the first study that assessed the relative risk of malignancy in patients with TAK compared to that in the general population using the nationwide population data. Due to the low prevalence of TAK, even the largest multi-center cohort only included 318 TAK patients^26,27^.

Only one previous study assessed the relative risk of malignancy in patients with TAK^22^; however, it used data from a single-center cohort. In addition, the study had 10 patients with overall malignancy out of the 180 patients with TAK, and it did not detect an increased risk of malignancy. In contrast, this study observed an increased risk of malignancy in patients with TAK compared to that in the general population. A reason for the different results could be an increased statistical power due to the larger number of patients.

This result is consistent with those of several other rheumatic diseases, including AAV. However, the finding is different from that for GCA, another type of large-vessel vasculitis. The mean age at the diagnosis of malignancy was 54 years old in this study. The late onset of GCA may be the reason for the absence of an increase in the relative risk of malignancy compared to that in the general population, given that the majority of GCA patients were over 70 years of age^28–30^.

A previous study reported an increased risk of MDS^22^. However, only one patient with MDS was included in that study; therefore, the results were inconclusive. In this study, we replicated the results of a previous study in which three MDS patients were identified, and the SIR also increased significantly. The occurrence of MDS in various types of vasculitis was reported. In a study of vasculitis-associated malignancies that included 60 patients with polyarteritis nodosa, leukocytoclastic vasculitis, AAV, and Henoch-Schönlein purpura, 21 patients were diagnosed with MDS, the most prevalent malignancy^31^. It is unclear whether the development of MDS is related to vasculitis or therapeutic agents such as cyclophosphamide^32,33^. Because patients with TAK who developed MDS in the previous study had a history of cyclophosphamide use, it was not possible to distinguish whether the occurrence of MDS was due to treatment^22^. In this study, no patients with MDS had a history of cyclophosphamide use. Therefore, TAK may be related to MDS.

The most frequent malignancy in this study was breast cancer, followed by thyroid cancer. There have been reports of breast cancer in patients with TAK^22,34^; however, none demonstrated an increased risk of breast cancer versus the general population. The large number of young female patients in this study may be one reason why breast and thyroid cancers were the two most common cancers.

Since few studies reported an increased risk of malignancies with immunosuppressive agents^35–37^, it is necessary to determine whether the development of malignancy is due to the disease or the treatment. No significant differences were observed in the use of immunosuppressive agents between TAK patients with malignancy and those without malignancy. The Cox proportional hazard analysis with medication usage as a time-varying variable was adjusted for the effects of age, sex, and index year, and it was performed for only glucocorticoid, azathioprine, and methotrexate users because other medications were used in three or fewer patients in the TAK with malignancy group. None of the medications were significantly related to the development of malignancy; therefore, the increased risk of malignancy was more likely due to the disease itself, rather than the medications. However, medications known to be more closely related to the development of malignancies, such as cyclophosphamides^38^, were not included in the analysis, which was a limitation of the present study.

Comorbidities of patients with TAK could be a confounding factor in this analysis. If the relative risk of malignancy in patients with TAK was calculated by matching with the control group according to the comorbidities in the patient group, more accurate results could be obtained. Unfortunately, we could only obtain information regarding the comorbidities from patients with TAK but not from the general population, because we retrieved data regarding the incidence of malignancies in the general population from the NCR database, which does not include information on comorbidities of each malignancy event. Instead, we calculated the SIRs of malignancies for patients with TAK with low/medium/high comorbidities and compared the risk of malignancy with that of the general population. In this analysis, the SIRs of malignancies were significantly increased in patients with medium and high comorbidities but not in those with low comorbidities. Thus, we could not exclude the confounding effect of comorbidities on the risk of malignancy in patients with TAK. Nevertheless, it would be difficult to draw clear conclusions from these results because we did not match the comorbidities between patients with TAK and the general population. Various comorbidities such as chronic obstructive pulmonary disease^39^ and diabetes mellitus^40^ are well-known risk factors for various types of malignancies; therefore, the finding that the number of malignancies increased with an increase in the number of comorbidities in this patient cohort is expected. We believe that a follow-up study using data with adjustments for comorbidities in the control group is needed to confirm these findings.

The cumulative incidence was checked to determine the period during which the risk of malignancy increased after the diagnosis of TAK. Considering that the average age was 54 years and the average duration between the diagnosis of malignancy and TAK was 3.38 years, the incidence of malignancy was expected to increase in the early phase of TAK diagnosis. However, we could not detect a period of high incidence of malignancy. Although it was impossible to obtain the disease activity information due to the nature of the data, the steady occurrence of malignancy throughout the observational period indirectly revealed that the malignancy occurrence in patients with TAK was not related to the disease activity or treatment. If we included patients who had a longer disease duration than those newly diagnosed between 2009 and 2019, it may demonstrate a different tendency.

To observe the effect of differences in medical systems and test methods according to the changes in time on the risk of malignancies, the patients were divided into three groups by the year of TAK diagnosis: 2009–2012, 2013–2016, and 2017–2019. The risk of overall malignancies was significantly higher in patients with TAK diagnosed between 2013–2016 and 2017–2019 but not in the 2009–2012 group. It is believed that the disease activity of TAK would be better controlled with a rapid diagnosis of TAK in the later period; therefore, these factors could not explain the increase in the number of cancer diagnoses between 2013 and 2019. It is difficult to identify the exact reason behind this finding based on the data of the present study alone; however, recent advancements in imaging techniques might be one of the reasons because newer imaging techniques allowed us to diagnose malignancies in the early stages.

The exact underlying mechanisms of increased malignancy risk in patients with vasculitis remain unclear; however, several potential mechanisms have been suggested. The most common explanation is that the risk of malignancy is increased due to a dysfunction in the immune system. Malignancy risk is vastly increased in immune deficiency conditions and immunosuppressed patients^41^. A normal immune system can act against tumor formation; however, dysregulation of the immune function in autoimmune diseases could potentially lead to cancer development^42^. Premature immunosenescence and excessive production of cytokines induce immunosuppression, and it may increase the risk of hematologic malignancies either directly or due to a chronic Epstein-Barr virus infection^43,44^. In the case of MDS and myeloproliferative disorders, decreased natural killer cell activity, phagocytic cell function, and dysregulated antigenic presentation with persistent immune stimulation were suggested as the underlying mechanisms^45–47^. In addition, chronic inflammation could mediate the development of malignancy^48,49^. The oxidative stress due to inflammation could damage the deoxyribonucleic acid, leading to cancer development by proto-oncogene activation, chromosomal rearrangement/amplification, and inactivation of the tumor suppressor genes^50^. Further research is needed on the increased risk of malignancy specific to TAK.

This study has several strengths. First, the subjects in our study consisted of all patients with TAK in South Korea, and the general population from NCR included virtually all populations of the country. Second, the study population and follow-up were well defined because of the broad coverage of the Korean NHIS. Practically, all diagnosed malignancies in South Korea can be detected using the NHIS.

This study has a few methodological limitations due to the inherent nature of health claims data. First, the data did not include information regarding the symptoms, laboratory results, and radiographic findings because the data were collected for administrative purposes rather than research. Therefore, the specific characteristics of the patients, including the disease severity, extent, and type, were unknown. Second, residual confounding by comorbidities cannot be excluded as previously described. Third, there might have been a surveillance bias because patients with TAK visited the hospital more frequently than the general population. We could not avoid this problem completely due to the retrospective nature of the study. However, some aspects of this study can indirectly infer the effect of surveillance bias. Usually, most patients undergo laboratory or imaging testing in the early stages of diagnosis. However, in this study, patients with TAK did not develop malignancies at the beginning of the follow-up but developed steadily as the study period progressed. In addition, when the index date was changed to that of the second clinical visit for sensitivity analysis, SIRs were still significantly increased in patients with TAK than that in the general population. Therefore, these findings might act as indirect evidence to show that the surveillance bias was not large in this study. In contrast, the incidence of cancer increased in patients diagnosed more recently based on the index date, and the reason might be due to the recent developments in the screening and imaging techniques, suggesting the possibility of a surveillance bias. Thus, prospectively designed follow-up studies are needed. Fourth, there might be discrepancies between the actual disease onset and diagnosis. The disease duration may have been underestimated because the time of symptom onset could not be determined. In a previous study, the time from onset to diagnosis was 2–11 years^51^. It could be an explanation of the relatively old age at diagnosis in this study. Finally, the mean observational period, 6.35 years, was relatively short to evaluate the occurrence of malignancy. That is because we only included newly diagnosed patients with TAK, and the available data were restricted from 2007 to 2020. If we could include more extensive data in the future, more conclusive results can be drawn.

An increased risk of hematologic and solid malignancies was observed in patients with TAK compared to that in the general population. No association was observed between the immunosuppressive agent use and malignancy. Malignancy occurred steadily throughout the study period. However, because of the limitations of the present study, the effects of comorbidity and surveillance bias could not be completely excluded. To the best of our knowledge, this is the largest study which investigated the risk of malignancy in patients with TAK.

## Supplementary Information

Supplementary Information 1.

Supplementary Information 2.

## Data Availability

The data that support the findings of this study will not be shared due terms of the contract signed with the Korean Health Insurance Review and Assessment Service that provided use with nationwide data in South Korea. According to these terms, the collected data must be discarded once the investigation has been concluded. Data are, however, available from the HIRA database (https://opendata.hira.or.kr/) by the appropriate data application processes.

## Abbreviations

AAV: Anti-neutrophil cytoplasmic antibody-associated vasculitis
CI: Confidence interval
GCA: Giant cell arteritis
HIRA: Health Insurance Review and Assessment Service
HR: Hazard ratio
ICD: International Classification of Diseases
MDS: Myelodysplastic syndrome
NCR: National Cancer Registry
NHIS: National Health Insurance Service
RID: Rare intractable disease
SIR: Standardized incidence ratio
TAK: Takayasu’s arteritis
