# Supplementary material for: Increased risk of malignancy in patients with Takayasu’s arteritis: a population-based cohort study in Korea
Source: Sci Rep. 2022 Dec 21;12:22047. doi: 10.1038/s41598-022-24324-0 (PMC9772336; doi:10.1038/s41598-022-24324-0)
Supplement: Supplementary file 1 — Supplementary Information 1. [file 41598_2022_24324_MOESM1_ESM.docx]

**Table S1.** List of comorbidities and their weights identified using the Charlson comorbidity index

| Comorbidities | ICD-10 | Original weight |
| --- | --- | --- |
| Myocardial infarction | I21.x, I22.x, I25.2 | 1 |
| Congestive heart failure | I09.9, I11.0, I13.0, I13.2, I25.5, I42.0, I42.5-I42.9, I43.x, I50.x, P29.0 | 1 |
| Peripheral vascular disease | I70.x, I71.x, I73.1, I73.8, I73.9, I77.1, I79.0, I79.2, K55.1, K55.8, K55.9, Z95.8, Z95.9 | 1 |
| Cerebrovascular disease | G45.x, G46.x, H34.0, I60.x-I69.x | 1 |
| Dementia | F00.x-F03.x, F05.1, G30.x, G31.1 | 1 |
| Chronic pulmonary disease | I27.8, I27.9, J40.x-J47.x, J60.x-J67.x, J68.4, J70.1, J70.3 | 1 |
| Rheumatologic disease | M05.x, M06.x, M31.5, M32.x-M34.x, M35.1, M35.3, M36.0 | 1 |
| Peptic ulcer disease | K25.x-K28.x | 1 |
| Mild liver disease | B18.x, K70.0-K70.3, K70.9, K71.3-K71.5, K71.7, K73.x, K74.x, K76.0, K76.2-K76.4, K76.8, K76.9, Z94.4 | 1 |
| Diabetes without chronic complication | E10.0, E10.1, E10.6, E10.8, E10.9, E11.0, E11.1, E11.6, E11.8, E11.9, E12.0, E12.1, E12.6, E12.8, E12.9, E13.0, E13.1, E13.6, E13.8, E13.9, E14.0, E14.1, E14.6, E14.8, E14.9 | 1 |
| Diabetes with chronic complication | E10.2-E10.5, E10.7, E11.2-E11.5, E11.7, E12.2-E12.5, E12.7, E13.2-E13.5, E13.7, E14.2-E14.5, E14.7 | 2 |
| Hemiplegia or paraplegia | G04.1, G11.4, G80.1, G80.2, G81.x, G82.x, G83.0-G83.4, G83.9 | 2 |
| Renal disease | I12.0, I13.1, N03.2-N03.7, N05.2-N05.7, N18.x, N19.x, N25.0, Z49.0-Z49.2, Z94.0, Z99.2 | 2 |
| Malignancy | C00.x-C26.x, C30.x-C34.x, C37.x-C41.x, C43.x, C45.x-C58.x, C60.x-C76.x, C81.x-C85.x, C88.x, C90.x-C97.x | 2 |
| Moderate or severe liver disease | I85.0, I85.9, I86.4, I98.2, K70.4, K71.1, K72.1, K72.9, K76.5, K76.6, K76.7 | 3 |
| Metastatic solid tumor | C77.x-C80.x | 6 |
| AIDS/HIV | B20.x-B22.x, B24.x | 6 |
| *ICD-10,* International Classification of Diseases, 10th revision code; *AIDS/HIV,* Acquired immunodeficiency syndrome/human immunodeficiency virus | | |

**Table S2.** Clinical characteristics of patients according to the presence or absence of malignancies

|  | TAK without malignancy | TAK with malignancy | *P*-value |
| --- | --- | --- | --- |
| n | 1,375 | 74 |  |
| Sex, female | 1120 (81.5) | 55 (74.3) | 0.17 |
| Age at diagnosis of TAK, years | 46.79 ± 15.55 | 50.93 ± 15.36 | 0.026 |
| Age at diagnosis of malignancy, years |  | 54.31 ± 15.20 |  |
| Duration of observation, years | 6.51 ± 3.17 |  |  |
| Duration until development of malignancy, years |  | 3.38 ± 2.86 |  |
| Medication |  |  |  |
| Glucocorticoids | 667 (48.5) | 35 (47.3) | 0.933 |
| Methotrexate | 450 (32.7) | 19 (25.7) | 0.256 |
| Azathioprine | 227 (16.5) | 10 (13.5) | 0.605 |
| Leflunomide | 20 (1.5) | 0 (0.0) | 0.594 |
| Cyclophosphamide | 12 (0.9) | 0 (0.0) | 0.882 |
| Mycophenolate mofetil | 24 (1.7) | 3 (4.1) | 0.322 |
| Tacrolimus | 34 (2.5) | 2 (2.7) | >0.999 |
| Cyclosporine | 30 (2.2) | 1 (1.4) | 0.945 |
| Anti-TNF | 17 (1.2) | 0 (0.0) | 0.683 |
| Tocilizumab | 7 (0.5) | 0 (0.0) | >0.999 |
| CCI |  |  | 0.066 |
| 0 | 277 (20.1) | 9 (12.2) |  |
| 1 | 390 (28.4) | 17 (23.0) |  |
| 2 | 308 (22.4) | 20 (27.0) |  |
| 3≤ | 177 (12.9) | 28 (37.8) |  |
| Comorbidities |  |  |  |
| Myocardial infarction | 39 (2.8) | 2 (2.7) | >0.999 |
| Congestive heart failure | 190 (13.8) | 10 (13.5) | >0.999 |
| Peripheral vascular disease | 19 (1.4) | 2 (2.7) | 0.669 |
| Cerebrovascular disease | 437 (31.8) | 28 (37.8) | 0.337 |
| Dementia | 11 (0.8) | 1 (1.4) | >0.999 |
| Chronic pulmonary disease | 433 (31.5) | 23 (31.1) | >0.999 |
| Rheumatologic disease | 177 (12.9) | 8 (10.8) | 0.735 |
| Peptic ulcer disease | 395 (28.7) | 22 (29.7) | 0.957 |
| Mild liver disease | 345 (25.1) | 26 (35.1) | 0.073 |
| Diabetes without chronic complication | 302 (22.0) | 30 (40.5) | <0.001 |
| Diabetes with chronic complication | 82 (6.0) | 6 (8.1) | 0.615 |
| Hemiplegia or paraplegia | 25 (1.8) | 2 (2.7) | 0.915 |
| Renal disease | 59 (4.3) | 2 (2.7) | 0.715 |
| Malignancy | 0 (0.0) | 0 (0.0) | NA |
| Moderate or severe liver disease | 4 (0.3) | 0 (0.0) | >0.999 |
| Metastatic solid tumor | 0 (0.0) | 0 (0.0) | NA |
| AIDS/HIV | 0 (0.0) | 0 (0.0) | NA |
| Numerical quantitative data are presented as mean ± standard deviation.  Categorical data are presented as frequencies (%).  *TAK,* Takayasu’s arteritis; *CCI*, Charlson comorbidity index; *TNF,* tumor necrosis factor; *AIDS/HIV* Acquired immunodeficiency syndrome/human immunodeficiency virus; *NA*, not applicable. | | | |

**Table S3.** Cox’s proportional hazard model analysis of medication usage and the occurrence of malignancy in patients with Takayasu’s arteritis, using each medication usage as a time-varying variable

|  | Crude HR | | Adjusted HR | |
| --- | --- | --- | --- | --- |
|  | HR (95% CI) | *p*-value | HR (95% CI) | *p*-value^†^ |
| Glucocorticoids | 1.08 (0.66–1.77) | 0.748 | 1.23 (0.75–2.01) | 0.406 |
| Methotrexate | 0.52 (0.13–2.13) | 0.366 | 0.51 (0.13–2.02) | 0.337 |
| Azathioprine | 0.87 (0.40–1.90) | 0.729 | 0.90 (0.41–2.01) | 0.801 |
| ^†^ *p*-values were derived from the Cox’s regression model, including glucocorticoid, methotrexate, and azathioprine use, as well as the age of patients at time of Takayasu’s arteritis diagnosis, sex, and the year of index date.  *HR,* hazard ratio; *CI,* confidence interval. | | | | |

**Table S4.** The SIRs of malignancies after categorizing the patients based on the number of comorbidities in patients with TAK and the general population

| Type of malignancy | Number of malignancies | Expected^¶^ | SIR (95% CI) |
| --- | --- | --- | --- |
| TAK with low comorbidity (CCI 0–1; n=683; 4574 PYs) | | | |
| All | 26 | 19.00 | 1.37 (0.89–2.01) |
| Solid malignancy | 24 | 18.19 | 1.32 (0.85–1.96) |
| Hematologic malignancy | 2 | 0.80 | 2.51 (0.30–9.07) |
| TAK with medium comorbidity (CCI 2; n=312; 1928 PYs) | | | |
| All | 18 | 9.69 | 1.86 (1.10–2.94) |
| Solid malignancy | 17 | 9.27 | 1.83 (1.07–2.93) |
| Hematologic malignancy | 1 | 0.41 | 2.43 (0.06–13.53) |
| TAK with high comorbidity (CCI ≥ 3; n=454; 2517 PYs) | | | |
| All | 30 | 16.6 | 1.81 (1.22–2.58) |
| Solid malignancy | 25 | 15.9 | 1.58 (1.02–2.33) |
| Hematologic malignancy | 5 | 0.75 | 6.69 (2.17–15.62) |
| ^¶^ Expected number of patients with malignancy calculated based on the incidence of malignancy in the age- and sex-matched general population.  *SIR*, standardized incidence ratios; *TAK*, Takayasu’s arteritis; *CI*, confidence interval; *PY*, person-year. | | | |

**Table S5.** The relative risk of malignancy stratified by the year of TAK diagnosis

| Type of malignancy | Number of malignancies | Expected^¶^ | SIR (95% CI) |
| --- | --- | --- | --- |
| Index date between 2009–2012 (n=520; 5080 PYs) | | | |
| All | 31 | 22.37 | 1.39 (0.94–1.97) |
| Solid malignancy | 28 | 21.41 | 1.31 (0.87–1.89) |
| Hematologic malignancy | 3 | 3.14 | 3.14 (0.65–9.16) |
| Index date between 2013–2016 (n=543; 3143 PYs) | | | |
| All | 30 | 16.57 | 1.81 (1.22–2.58) |
| Solid malignancy | 26 | 15.85 | 1.64 (1.07–2.40) |
| Hematologic malignancy | 4 | 0.71 | 5.61 (1.52–14.36) |
| Index date between 2017–2019 (n=386; 974 PYs) | | | |
| All | 13 | 5.21 | 2.50 (1.33–4.27) |
| Solid malignancy | 12 | 4.97 | 2.41 (1.25–4.21) |
| Hematologic malignancy | 1 | 0.23 | 4.33 (0.11–24.11) |
| The patients were divided into three groups according to the time of TAK diagnosis: 2009–2012, 2013–2016, and 2017–2019.  ^¶^ Expected number of patients with malignancy calculated based on the incidence of malignancy in the age- and sex- matched general population.  *SIR*, standardized incidence ratios; *TAK*, Takayasu’s arteritis; *CI,* confidence interval; *PY,* person-year. | | | |
