# Supplementary figures and images for: Increased risk of malignancy in patients with Takayasu’s arteritis: a population-based cohort study in Korea
Source: Sci Rep. 2022 Dec 21;12:22047. doi: 10.1038/s41598-022-24324-0 (PMC9772336; doi:10.1038/s41598-022-24324-0)

Figure S1. Cumulative incidence of malignancy during observation period

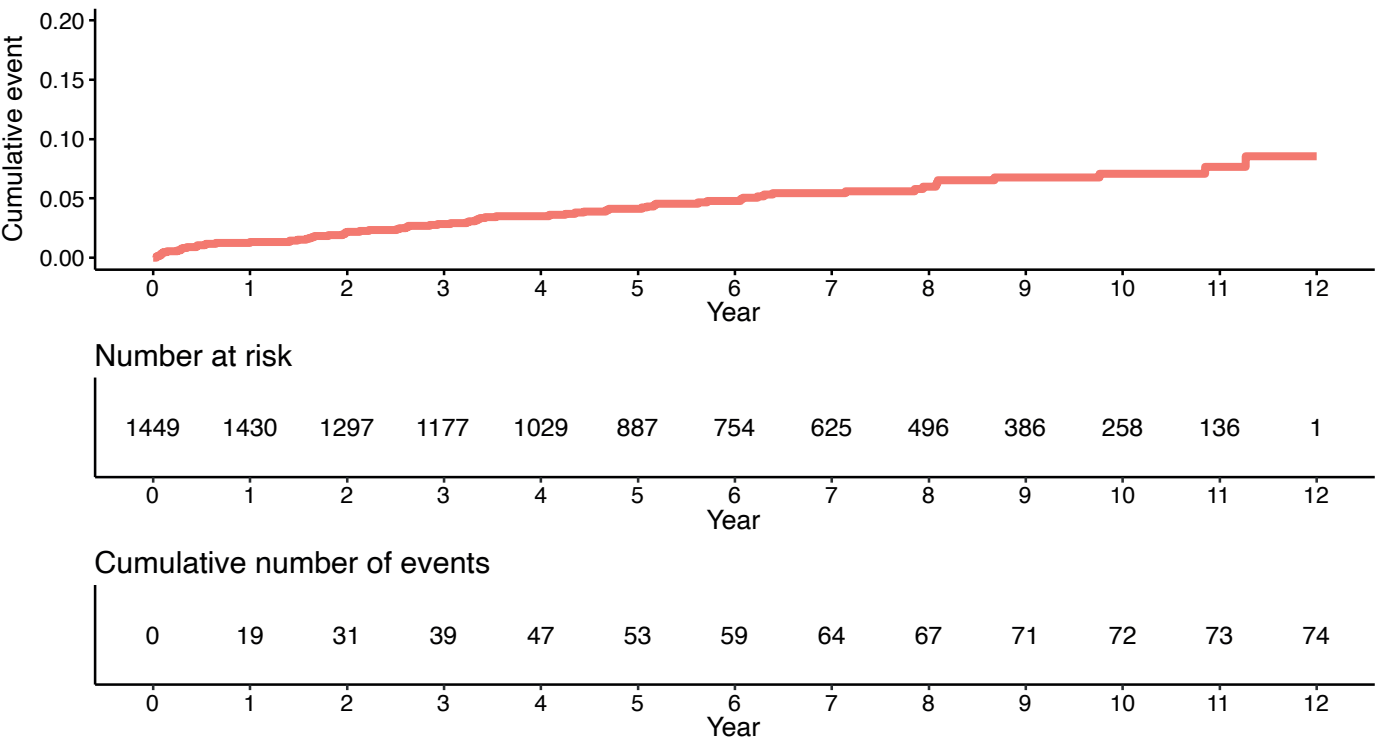

Supplement: Supplementary file 2 — Supplementary Information 2. [file 41598_2022_24324_MOESM2_ESM.pdf]
